# Predicting Field Effectiveness of Endophytic *Bacillus subtilis* Inoculants for Common Bean Using Morphometric and Biochemical Markers

**DOI:** 10.3390/plants13131769

**Published:** 2024-06-26

**Authors:** Oksana Markova, Svetlana Garipova, Aelita Chistoedova, Viktoriia Matyunina, Alsu Lubyanova, Oksana Lastochkina, Arsenii Garipov, Irina Shpirnaya, Lyudmila Pusenkova

**Affiliations:** 1 Institute of Nature and Human, Ufa University of Science and Technology, Zaki Validy Str. 32, 450076 Ufa, Russia; o-ksana@list.ru (O.M.); achistoyedova@mail.ru (A.C.); viktoria030700@mail.ru (V.M.); arsenygaripov2000@gmail.com (A.G.); i-shia@yandex.ru (I.S.); 2 Institute of Biochemistry and Genetics—Subdivision of the Ufa Federal Research Center of the Russian Academy of Sciences, Pr. Oktyabrya 71, 450054 Ufa, Russia; lubyanova555@mail.ru (A.L.); oksana.lastochkina@ufaras.ru (O.L.); 3 Bashkir Research Institute of Agriculture—Subdivision of the Ufa Federal Research Center of the Russian Academy of Sciences, R. Zorge Str. 19, 450059 Ufa, Russia; l.pusenkova@mail.ru

**Keywords:** *Phaseolus vulgaris* L., seed productivity, malondialdehyde, hydrogen peroxide, catalase, peroxidase, superoxide dismutase, correlation relationships

## Abstract

According to four field experiments, after the inoculation of *Phaseolus vulgaris* L. cultivar Ufimskaya with the commercial strain *Bacillus subtilis* 26D and the promising strain *B. subtilis* 10–4, it was found that inoculation with *B. subtilis* 10–4 improved seed productivity (SP) by 31–41% per plant, but only in dry years. In contrast, all 4 years of inoculation with *B. subtilis* 26D were ineffective or neutral. It was intended to determine the growing and biochemical characteristics of inoculated 7-day-old plants, which correlate with the field SP of bacterial preparations. The SP of inoculated plants (average of 4 years) correlated with root length (0.83), MDA content (−0.98), and catalase (CAT) activity in roots (−0.96) of week-old seedlings. High correlation coefficients between the H_2_O_2_ content in the roots and SP (0.89 and 0.77), as well as between the H_2_O_2_ content in shoots and SP (0.98 and 0.56), were observed only in two dry years, when the influence of bacteria was detected. These physiological indicators were identified as potential markers for predicting the effectiveness of the endophytic symbiosis between bean plants and *B. subtilis* strains. The findings may be used to develop effective microbial-based, eco-friendly technologies for bean production.

## 1. Introduction

Beans (*Phaseolus vulgaris* L.) are a vital component of sustainable agriculture and food security, being the world’s major legume crop used directly for human consumption [1]. Global bean production reached 27.5 million metric tons in 2020, grown on a global area of approximately 34.8 million hectares [2]. Bean seeds are rich in minerals and unsaturated fatty acids, such as linoleic and oleic acids, which help to protect against obesity, strengthen the immune system, and prevent cholesterol. These omega 6 and omega 9 fatty acids, in addition to being physiologically and biochemically essential for body functions, also play an important role in healthy tissue development [3]. Nutritionists characterize beans as an exceptional nutritional resource due to their high protein content and combination of carbohydrates, dietary fiber, and minerals, especially iron and zinc [4]. Bean plants, like other legumes, have a beneficial effect on soil fertility and health by means of symbiotic nitrogen fixation [5,6], protecting the soil from erosion as green manure, suppressing weeds, and conserving soil moisture [7]. 

In an increasingly warming climate, water stress is a serious environmental problem that reduces crop yields. The common bean is sensitive to soil moisture deficiency in the period from the beginning of germination to the budding of plants and during the period when fruits begin to form [8,9,10]. Stressful conditions also reduce the ability for symbiotic nitrogen fixation of plants. One of the important strategies for improving food security and maintaining soil fertility is breeding bean cultivars resistant to drought conditions [11,12] and capable of a high level of nodule-forming activity [13,14]. The ability of plants to tolerate unfavorable environmental conditions can be enhanced by inoculation with highly effective strains of endophytic bacteria (EB) [15,16,17].

Endophytes, as a ubiquitous associate of the plant, play a pivotal role in the regulation of the primary and secondary metabolism in their host plant [18,19]. In addition, growth-promoting bacteria are components of soil ecosystems and do not cause environmental pollution. They are able to synthesize hormones and physiologically active substances that improve plant nutrition and protection from phytopathogens and abiotic stresses [20,21,22]. Other than phytohormones, reactive oxygen species (ROS), Ca^2+^, NO_2_, systemin, and inositol phosphates also work as signaling agents in plants [23]. In response to endophyte colonization, plants synthesize osmoprotectants, such as glutamate, glycine betaine, K^+^, proline, trehalose, and ectoine, which helps them cope with abiotic stress [24]. Involvement of endophytes in the physiological processes of plants stabilizes subcellular structure and scavenges free radicals to manage cytoplasmic osmotic potential [25]. 

Currently, biological products based on EB are used to increase the productivity and adaptive potential of agricultural plants [18]. However, the symbiotic interactions between plants and endophyte may result in different outcomes as defined by the fitness benefits of each of the partners [26]. Under stressful conditions, EB complement the physiological activity of plants by synthesizing useful metabolites, whereas under normal conditions, a self-sufficient adaptive variety can survive without “helpers” [15]. For example, under non-stress conditions, the inoculation of common beans with *Rhizobium tropici* and *Azospirillum brasilense* did not influence the leaf area, root dry matter and volume, or total dry matter. However, under moderate and severe drought stress, the inoculation and the co-inoculation with these bacteria resulted in higher root volume, leaf area, and total dry matter when compared to non-inoculated plants [27]. 

Endophytes are known to have flexible relationships in microbe–host–environment interactions across spatial and temporal scales [28] and contain genetic information that encodes new traits in the host plant [29]. If any imbalance occurs in the host–microbe interaction, endophytes can change their lifestyle from mutualistic to pathogenic [30,31,32]. Zolotistaya bean cultivar, inoculated with endophytic strains *Bacillus subtilis* 26D, increased seed productivity by 39% in comparison to the control without inoculation, but when it was inoculated with *Bacillus subtilis* 10–4, no significant differences compared to the control were found. At the same time, inoculation of Ufimskaya bean cultivar with *Bacillus subtilis* 26D was ineffective and inoculation with *Bacillus subtilis* 10–4 promoted an increase in seed productivity by 31% [33]. In another experiment with these cultivar–strain combinations, both strains were effective with the Zootistaya cultivar, but *Bacillus subtilis* 26D was ineffective with the Ufimskaya cultivar [34]. Ufimskaya cultivar was more adaptive to the contrasting environmental conditions than the Zolotistaya cultivar [35]. Ufimskaya bean cultivar was capable of resisting the effects of short-term stress (a 48-h exposure of plants to 1% NaCl). Despite the effect of the stressor in plants without inoculation, the water status of the roots improved, at the same time the inoculation, depending on the strain, had a weak (strain 10–4) or negative (strain 26D) effect on the water content of the roots [36]. 

The genus Bacillus is one of the most widespread representatives of the endophytic plant community, which can ameliorate water stress tolerance in maize and common bean [8] and produce antimicrobial substances against phytopathogens, phytohormones, and siderophores that improve plant growth and the availability of nutrients [21]. Inoculation with the commercial endophytic strain *B. subtilis* 26D improved the productivity, disease and stress resistance of wheat plants [37,38,39], awnless brome (*Bromopsis inermis* L.), pea (*Pisum sativum* L., cv. Chishminskii 95), maize (*Zea mays* L., cv. RIK-340) [39], common bean [40], potato [41], and beetroot [42]. Strains of EB *B. subtilis* 26D and 10–4 have a set of properties that stimulate plant growth; they are capable of nitrogen fixation, and producing indolyl-3-acetic acid (IAA) and siderophores; strain *B. subtilis* 26D was capable of solubilizing phosphates [40]. 

Despite the fact that EB preparations do not exhibit strict species specificity [18], the effect of inoculation depends on the genotype of the host plant [17,33,34,38,39,43] and doses of introduced bacteria [44]. For effective plant cultivation, it is important to address the possibilities of controlling plant productivity using the potential of EB and the limiting factors constraining this process [15,16,17,18]. According to recent studies, it is necessary to select the strain–dose combination individually for each plant genotype [17,18,44]. In order to conduct such screening, it is important to determine the phenotypic traits of the inoculated plants in the early stages of ontogenesis that correlate with the field effectiveness of bacterial preparations. They would serve as markers for the effective and ineffective pairing of the symbionts of inoculated plants. 

The purpose of this study was to analyze the interaction of certain cultivar–strain and dose-dependent combinations of EB *B. subtilis* with bean plants in different agroecological conditions and to determine the growing and biochemical characteristics of inoculated 7-day-old plants, which correlate with the field seed productivity of bacterial preparations. 

## 2. Results

### 2.1. The Influence of Bean Seed Inoculation with Endophytic Bacterial Strains on the Physiological Characteristics and Productivity of Plants under Field Conditions

The effect of inoculation of beans with endophytic bacteria *B. subtilis* 26D and 10–4 on seed productivity was studied in 4 field experiments (Table 1). Under moisture deficiency conditions (2018 and 2023) bean plants of the Ufimskaya cultivar, inoculated with the *B. subtilis* strain 10–4, increased the seed productivity per plant by 31–41%, and per square meter increased by 19–20% in comparison to the control plants that were not inoculated. However, in the years, when agroclimatic conditions were relatively favorable (2020 and 2021), the effect of inoculation with this strain on seed productivity was neutral, similar to the control group. On the other hand, inoculation with the *B. subtilis* strain 26D resulted in a decrease in the productivity of this particular bean cultivar, regardless of the weather conditions in all studied years.

Strain 10–4 in the preparation was used at a dose of 10^5^ cells mL^−1^, since in preliminary experiments it had a stimulating effect on the growth of bean plants [33,40]. Strain 26D was introduced at a dose of 10^8^ cells mL^−1^ according to the manufacturer’s recommendation, but in 2020 a low dose (10^5^ cells mL^−1^) of this strain was also tested [34]. Since the phenotypic characteristics of inoculated plants in 2018 and 2020 were described earlier [33,34], in this study the results of the 2023 field experiment were examined in detail (Table 2, Table 3, Table 4 and Table 5). 

In 2023 (HTC 0.6), in the variant without inoculation, bean germination on the 9th day was 47% and before the budding phase it increased to 67% (Table 2). Bacterial treatment of seeds improved germination rates at the first time point of measurement to 57–73%, at the second point to 77–90%. During the period of bean ripening in 2023, due to the impact of unfavorable weather conditions on the plants, the plant survival rate in the control variant did not exceed 70%. The use of endophytic strains increased the survival of plants in all experimental variants to 75–87%. Inoculation had a positive effect on plant height only when treated with strain 10–4 at a dose of 10^5^ cells mL^−1^: in the budding phase the difference with the control was 17%, in the ripening phase—7%.

Pretreatment of bean seeds with strains of EB further contributed to the acceleration of plant development (Table 3). In the variants of treatment with strain 10–4 with both doses, the proportion of plants in the flowering phase was 2 times greater, and in full ripeness phase this difference was 2.7–3 times compared to control plants. The proportion of plants that reached the stage of full ripeness was equal in the variants of inoculation with strain *B. subtilis* 26D (10^8^ cells mL^−1^) and 10–4 (10^5^ cells mL^−1^), significantly ahead of control without inoculation. The lag from other options was noticeable during inoculation with 26D (10^5^ cells mL^−1^), but this rate of maturation was faster than control.

Indicators of plant water status were determined at the beginning of bean ripening during the period of the greatest intensity of extreme climatic conditions after a long drought (Table 4). In terms of leaf total water content (W), inoculated plants differed slightly from the control. The water-holding capacity (R) in the leaves of plants inoculated with the *B. subtilis* strain 26D at a dose of 10^5^ cells mL^−1^ was 2 times higher than the control. When pre-treating seeds with *B. subtilis* 26D at a dose of 10^8^ cells mL^−1^, *B. subtilis* 10–4 at a dose of 10^5^ cells mL^−1^ and at a dose of 10^8^ cells mL^−1^, it exceeded the control values by 37%, 47% and 58%, respectively, compared to the control. In the same experimental variants, an increase in relative water content (RWC) was noted by 18%, 9% and 14%, respectively, relative to control plants.

Analysis of the elements of the crop structure showed that treatment of the Ufimskaya variety with the *B. subtilis* 10–4 strain in both doses contributed to an increase in the number of beans by 33 and 39%, the number of seeds by 35 and 65%, and the weight of seeds by 42 and 58% compared to control plants without inoculation. Treatment with *B. subtilis* strain 26D at both doses was ineffective (Table 5).

### 2.2. The Influence of Bean Inoculation with Strains of Endophytic Bacteria on the Growth and Antioxidant Status of 7-d-Old Plants in Laboratory Conditions (In Vitro)

**Figure 1:**
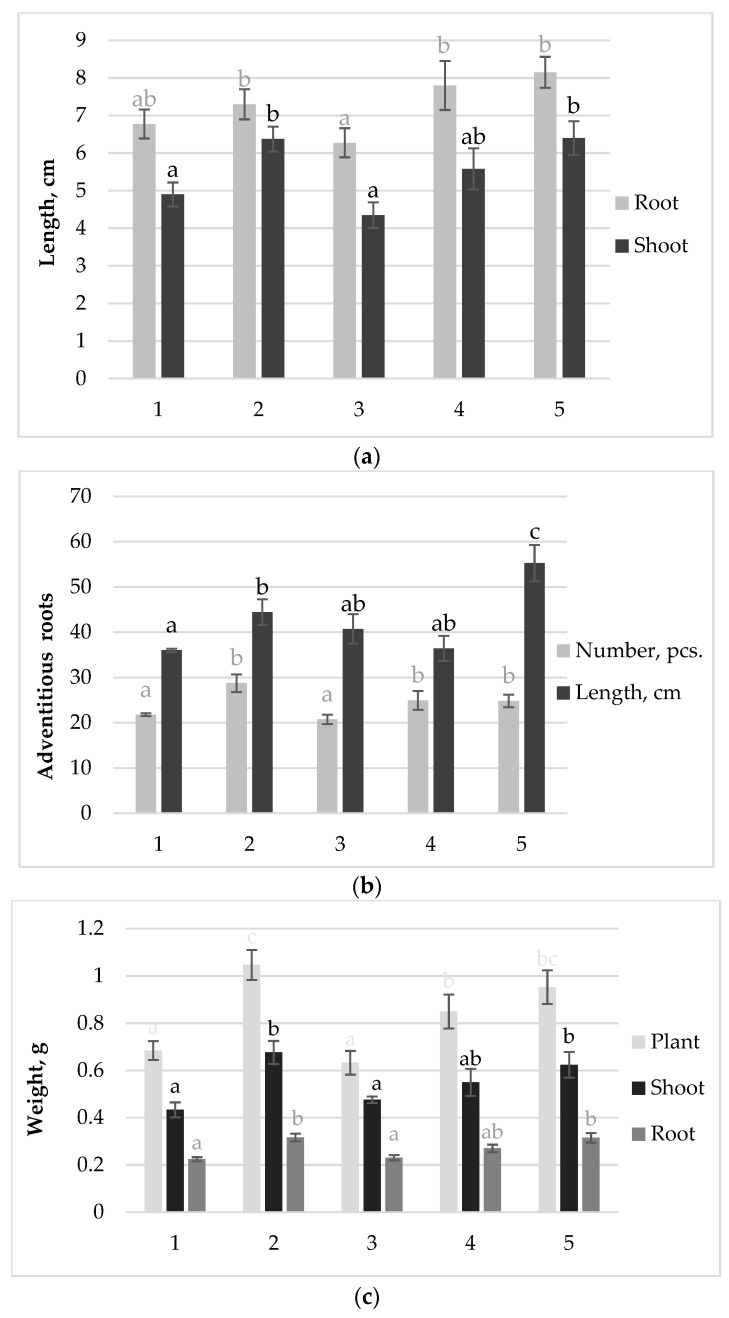
Growth parameters of 7-d-old bean seedlings treated with different doses of *B. subtilis* strains: (**a**) root and shoot length; (**b**) number and sum of adventitious root lengths; (**c**) fresh weight of plant, shoot and root; 1—Control without inoculation; 2—*B. subtilis* 26D × 10^5^ cells mL^−1^; 3—*B. subtilis* 26D × 10^8^ cells mL^−1^; 4—*B. subtilis* 10–4 × 10^5^ cells mL^−1^; 5—*B. subtilis* 10–4 × 10^8^ cells mL^−1^. The bars indicate the mean values of 20 plants ± SEM. Different letters indicate a significant difference between the means at the level of *p* < 0.05.

To predict effective and ineffective combinations of a variety with different strains of EB, it is important to determine the morpho-physiological markers of plants at the initial stages of symbiosis development, which are closely related to the production characteristics of plants. For this purpose, the growth performance of 7-d-old plants inoculated with different dose-strain combinations was analyzed in vitro. Inoculation with strain 10–4 in low and high doses contributed to an increase in plant root length by 15% and 20%, respectively, as well as an increase in shoot length by 14% and 30%, respectively, compared to the non-inoculated control (Figure 1a). The number of adventitious roots in the same experimental variants increased on average by 14% relative to the control (Figure 1b). When inoculated with strain 10–4 at a high dose, the total length of adventitious roots increased by 55% compared to the control. The weight of plants inoculated with strain 10–4 in low and high doses was 14% and 42% greater than in the control (Figure 1c). With these treatments with strain 10–4, an increase in plant weight by 25 and 40% was noted in both low and high doses respectively, compared to the control. When inoculated with strain 26D at a high dose, which was ineffective in four field trials, all measured growth parameters were at the level of control bean plants without inoculation. Reducing the dose of 26D to 10^5^ cells mL^−1^ had a positive effect on the length of the shoot, the number and length of adventitious roots, and the weight of the root and shoot of plants. Therefore, by applying the optimal dose, it is possible to reduce the adverse effects of the strain on the growth processes of bean plants.

**Figure 2:**
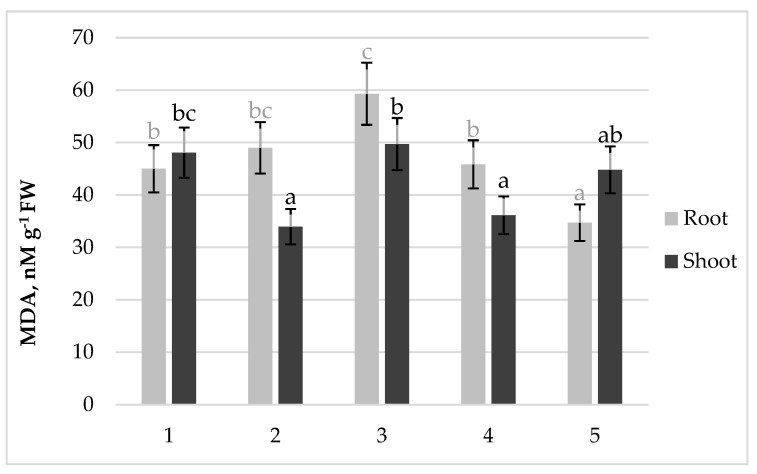
Effect of different doses of *B. subtilis* strains on the content of malondialdehyde (MDA) in the roots and shoots of 7-day-old bean plants: 1—Control; 2—*B. subtilis* 26D × 10^5^ cells mL^−1^; 3—*B. subtilis* 26D × 10^8^ cells mL^−1^; 4—*B. subtilis* 10–4 × 10^5^ cells mL^−1^; 5—*B. subtilis* 10–4 × 10^8^ cells mL^−1^. The bars indicate the mean values of three repetitions ± SEM (*n* = 3). Different letters indicate a significant difference between the means at the level of *p* < 0.05.

**Figure 3:**
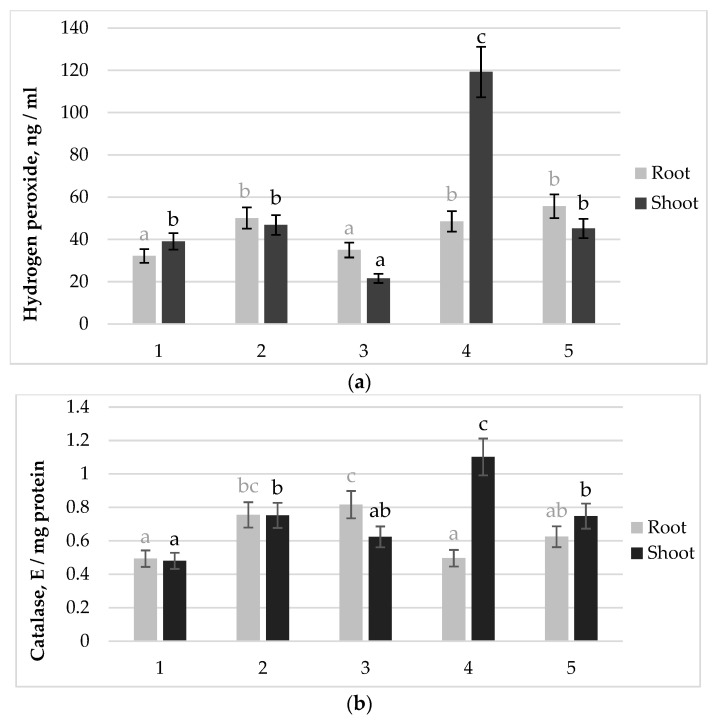

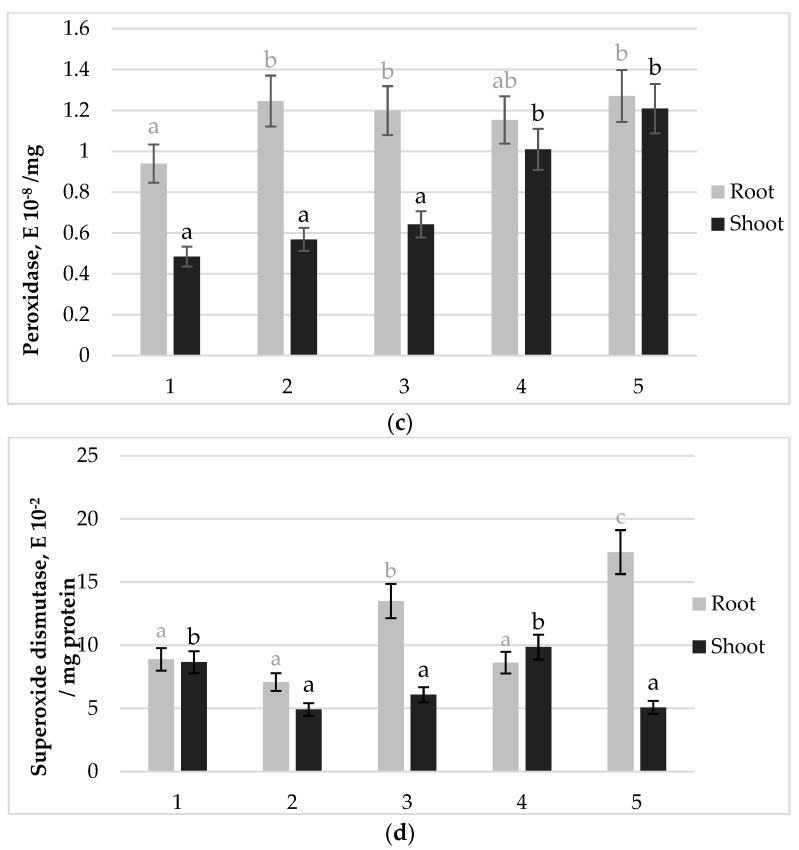
Effect of different doses of *B. subtilis* strains on the content of (**a**) hydrogen peroxide (H_2_O_2_), (**b**) catalase (CAT), (**c**) peroxidase (POD) and (**d**) superoxide dismutase (SOD) activity in the roots and shoots of 7-day-old bean plants: 1—Control; 2—*B. subtilis* 26D × 10^5^ cells mL^−1^; 3—*B. subtilis* 26D × 10^8^ cells mL^−1^; 4—*B. subtilis* 10–4 × 10^5^ cells mL^−1^; 5—*B. subtilis* 10–4 × 10^8^ cells mL^−1^. The bars indicate the mean values of three repetitions ± SEM (*n* = 3). Different letters indicate a significant difference between the means at the level of *p* < 0.05.

To clarify the nature of the physiological processes occurring in inoculated plants, the biochemical parameters of the antioxidant status in the roots and shoots of week-old plants were determined: the content of H_2_O_2_, MDA (Figure 2) and the activity of oxidoreductases: CAT, POD and SOD (Figure 3). To understand the intensity of the work of the antioxidant system, the coefficients of the ratio of the activity of each of the oxidoreductases to the content of H_2_O_2_ were calculated (Table 6). It was of interest to analize the indicators of redox status in the experimental variant that was ineffective for increasing germination, biomass, and bean yield (strain 26D at a dose of 10^8^ cells mL^−1^) compared with other inoculation variants.

In the ineffective inoculation option (26D, 10^8^ cells mL^−1^), a higher accumulation of MDA was observed in roots compared to the control and other inoculation options (Figure 2). When inoculated with a low dose of the same strain (26D, 10^5^ cells mL^−1^), which had a neutral effect on the final seed productivity, the MDA content in the roots of pretreated plants was comparable to the control, and in the shoots, it was even lower than the control. In the effective treatment options (strain 10–4 in both doses), the MDA level in roots was lower than in the ineffective option (strain 26D, 10^8^ cells mL ^−1^), and in the most effective treatment option (strain 10–4 at a dose of 10^8^ cells mL^−1^), the MDA content in the roots was significantly lower than in control. 

Another important indicator of the redox status of plants treated with biological preparations of different effectiveness was the level of H_2_O_2_ in tissues (Figure 3a). It is important that in the ineffective variant of inoculation (26D, 10^8^ cells mL^−1^), a low H_2_O_2_ content was noted in the roots and shoots. Moreover, the use of a small dose of the same strain (26D, 10^5^ cells mL^−1^) contributed to an increase in the H_2_O_2_ content in the roots and shoots by 56% and 46%, respectively, compared to the control. The H_2_O_2_ level in shoots was the highest (3 times higher than the control) when treated with the effective strain 10–4 at a dose of 10^5^ cells mL^−1^. The use of this strain at a high dose of 10^8^ cells mL^−1^ led to a moderate increase in the H_2_O_2_ content in roots and shoots by 71% and 15%, respectively, relative to the control, comparable to the increase the H_2_O_2_ content in variant of inoculation with 26D at a dose of 10^5^ cells mL^−1^.

Inoculation with effective strain-dose combination (10–4, 10^5^ cells mL^−1^) resulted in 2.2- and 2-fold increase in the activities of CAT (Figure 3b) and POD (Figure 3c) in plant shoots, respectively, compared to the control. SOD activity in shoots in this experimental variant also increased relative to other inoculation variants to the control level (Figure 3d). In the ineffective variant of inoculation with the strain (26D, 10^8^ cells mL^−1^), a moderate (by 20%) increase in the activity of CAT and POD, as well as a decrease in SOD activity (by 30%) compared to the control, was observed in shoots with a reduced level of H_2_O_2_ content. Analysis of bean roots exposed to pre-sowing treatment with strain 26D at a dose of 10^8^ cells mL^−1^ showed that the H_2_O_2_ content was at the control level, and the activity of CAT, POD, and SOD increased by 67%, 29% and 56%, respectively, relative to control plants.

Comparing the total activity of each enzymes in roots + shoots (Figure 3), it can be noted that the total activity of CAT in all inoculated variants of the experiment regardless of the degree of effectiveness of the biologics was increased by 1.5 times compared to the non-inoculated control; the total activity of POD was higher (especially in the shoots) in plants inoculated with effective strain 10–4, compared to ineffective strain 26D; total SOD activity in the plant (especially in the roots) reflected a tendency to increase the enzyme activity when inoculated with a higher dose of the biological of each strain relative to its low dose.

The features of the redox status in the ineffective variant of inoculation are not visible, based on the overall activity of enzymes, but when recalculating the specific activity of enzymes per unit of H_2_O_2_ contained in the tissues (SOD/H_2_O_2_, CAT/H_2_O_2_, and POD/H_2_O_2_), there is a noticeable increase in the intensity of the work of oxidoreductases specifically in ineffective variant of the experiment (strain 26D at a dose of 10^8^ cells mL^−1^): the coefficients of specific activity of the enzymes SOD, CAT/H_2_O_2_, and POD in the roots increased by 40%, 53% and 17%, and in the shoots—by 27%, 91% and 15%, respectively, compared to the control (Table 6). In effective variants of inoculation with strain 10–4 in low and high doses, the coefficients of specific activity of all or almost all oxidoreductases were reduced in both roots and shoots compared to control plants.

### 2.3. Analysis of the Correlation Relationships between the Activity of the Antioxidant System of 7-d-Old Plants In Vitro and the Final Productivity of Bean Plants Inoculated with Appropriate Strain-Dose Combinations of Endophytic Bacteria

To identify markers of the effectiveness of biological preparations, which in the early stages of plant development could predict the mutualistic or antagonistic nature of the formation of endophytic relationships, morphometric parameters, biochemical properties of 7-day inoculated plants in laboratory conditions, were compared with the final field seed productivity. The correlations between the compared traits were analyzed both under conditions of the positive effect of the drug and under conditions when the effect of the drug on seed productivity was neutral or negative (Table 7).

The average weight of seeds per plant closely correlated with the root length of week-old plants (r = 0.83). Moreover, the significance of this feature was higher in the years with a HTC of 0.6. The correlation coefficients were 0.99 and 0.94 in the experiments of 2018 and 2023. In relatively favorable conditions (2020 and 2021), the relationship between root length from week-old plants and final productivity in the corresponding inoculation varieties was significantly weaker. The other growth indicators of 7-d-old plants were characterized by large variability of correlation coefficients in different years, which reduces their diagnostic significance.

Biochemical indicators of inoculated one-week-old plants in vitro, closely correlating with the field efficiency of inoculation, were reduced MDA content in the roots compared to the control, reduced CAT activity in the roots and increased levels of H_2_O_2_ and CAT in the shoots. It is proposed to take these indicators as the basis for assessing the effectiveness of symbiosis in the early stages of plant ontogenesis and to evaluate their validity in subsequent studies.

## 3. Discussion

The Ufimskaya cultivar selected for the experiment produced a yield of up to 12–15 g per plant depending on the sowing time under favorable conditions [45]. But in years with extremely low moisture availability, the seed productivity of the cultivar decreased by 2–4 times compared to optimal hydrothermal conditions [35]. In a four-year experiment, the level of seed productivity of the Ufimskaya cultivar under different hydrothermal conditions corresponded to the average long-term data for the period 2003–2023 and ranged from 3.6 to 12.3 g per plant (Table 1). It was assumed that through inoculation with growth-stimulating bacteria it would be possible to increase the resistance of plants to moisture deficiency.

Both of the selected strains of EB *B. subtilis* 26D and 10–4 performed the plant growth-promoting traits and ensured viability and turgor during a further 8 days of exposure of the plants to a solution of 2% NaCl due to an increase in the content of proline and the strengthening of the cell walls under lignification [40]. But in the presented 4-year field experiments with the Ufimskaya cultivar, inoculation with the *B. subtilis* 10–4 strain had a positive effect on seed productivity only under moisture deficiency (HTC 0.5–0.6). Under relatively favorable conditions, it did not provide an advantage in yield compared to the control. At the same time, strain 26D turned out to be ineffective in increasing the seed productivity of inoculated plants in all experiments (Table 1).

The issue that the effectiveness of the use of EB-based preparations may depend both on the genotypes of the plant and bacteria, their signaling interactions, and their environmental conditions is discussed in the literature [16]. For example, several isolates of EB that stimulated the growth of many types of agricultural crops had a positive effect on seedling root growth only in one of the two oat cultivars. In the other cultivar, the bacterial isolates had either no effect or a negative effect on root growth [43].

Under normal and stress conditions, the cultivar–strain specificity can manifest itself differently. For example, under non-stress conditions, inoculation of the Estilo cultivar bean plants with the AP-3 strain led to an increase in the dry weight of shoots and roots compared to non-inoculated plants; inoculation with the PRBS-1 strain resulted in a neutral effect but under water stress conditions, both strains did not cause a significant difference in shoot and root dry weights compared to non-inoculated plants [8]. In experiments with bean plants grown under different conditions of moisture supply, it was shown that the positive effect of EB compared to the non-inoculated control, on the contrary, was more significant when moisture deficiency increased than under normal conditions [46]. Using a meta-analysis to summarize the 52 published articles on the effects of plant growth-promoting rhizobacteria (PGPR) on root biomass, shoot biomass, and yield under well-watered and drought conditions, it was found that the effect of PGPR was significantly greater under drought conditions than under normal conditions [47]. This is also consistent with the results obtained in this study. The Ufinskaya cultivar’s own high resistance to short-term and weak drought was demonstrated in the model experiment [36]. In relatively normal moisture field conditions (HTC 0.9–1.1 in 2020 and 2021), cultivar resistance had a more significant influence on the formation of productivity than the influence of bacterial inoculation. But under conditions of severe stress (HTC 0.5–0.6 in 2018 and 2023), the inoculation with *B. subtilis* 10–4 turned out to be significant.

Experiments revealed a negative effect of inoculation with strain 26D at a high dose on the seed productivity of plants. To understand the reason for this, a comparison was made of the various characteristics of inoculated plants under field conditions in 2023. The analysis of growth indicators, survival, phenology, water status, and yield structure of inoculated plants in field experiments showed that the main properties that differ in the ineffective symbiosis of the Ufimskaya cultivar with strain 26D from the effective symbiosis of this cultivar with the strain 10–4 were the lower plant height and the lower number of formed generative organs, the pods, which had resulted in the production of a smaller number and weight of seeds (Table 1, Table 2, Table 3, Table 4 and Table 5). In terms of germination, survival, speed of development, and resistance to water deficiency in the plants of other experimental variants differed little from the ineffective variant of inoculation with strain 26D at a high dose. Thus, the inoculation with strain 26D limited the formation of the generative organ number of the plant, not due to instability due to drought nor the inhibition of development but perhaps due to ineffective redistribution of nutrients between vegetative and generative organs.

To understand the factors limiting generative productivity, we turned to the properties of these strains, manifested in vitro. It is currently known that *B. subtilis* 26D produces 2 times less amount of auxin from tryptophan in vitro than *B. subtilis* 10–4 [40]. This may be important information for the formulation of a hypothesis, because a two-vertex dependence of root and shoot growth of week-old plants in response to seed priming in a gradient of concentrations of exogenous heteroauxin was revealed for the Ufimskaya cultivar [48]. The assumption appeared that the reason for the ineffectiveness of strain 26D at a high dose (10^8^ cells mL^−1^) could be its production of auxin in a non-optimal amount, which just hit the pessimum of the concentration curve. The relationship between super-optimal IAA production and the dose-dependent effects of strains on the growth of pea plants and on the yield of inoculated plants was reported in a study [44]. Further studies aimed at identifying this dose-dependent factor would be of interest.

The connection between the production of auxin by bacteria, which acts as a signaling molecule associated with the immune system, has been found in several studies. During the colonization of beneficial bacterium *Bacillus velezensis* in *Arabidopsis thaliana*, a feedback loop is established: bacterial colonization triggers an immune reaction and production of ROS, which, in turn, stimulate auxin production by the bacteria. Auxin ensures bacterial survival and efficient root colonization, allowing the bacteria to inhibit fungal infection and promote plant health [49]. In a study of Arabidopsis, the simultaneous application of H_2_O_2_, as an inducer of ROS, and auxin altered the shape of the root system architecture in a dose-dependent manner, acting as a fine-tuning mechanism for auxin-derived developmental gradients [50]. Analysis of gene expression in cotton seedling roots, inoculated with growth promoting bacterium *B. amyloliquefaciens*, showed that the most up-regulated genes related to auxin pathways, nitrate assimilation, and the production of a variety of antioxidant enzymes [29]. This up-regulation of genes fit into the rhizophagy hypothesis, according to which microbes alternate between the root intracellular endophytic phase and the free-living soil phase. Microbes assimilate soil nutrients in the free-living phase, which are extracted through exposure to reactive oxygen produced by the host plant in the intracellular endophytic phase [51,52]. To protect themselves from superoxide, endophytes produce antioxidants, including nitric oxide, to denature superoxide. These two chemical interactions between endophytes and plant cells represent the key exchange of carbon and nitrogen nutrients between symbionts [53]. If the microbe is highly resistant to reactive oxygen (superoxide), which is used in plant root cells to control and extract nutrients from internalized microbes, then interference with the endomicrobiome can occur, increasing stress and reducing plant fitness, causing stunted growth and reduced nutrient uptake plants [16].

It can be assumed that the ineffectiveness of *B. subtilis* 26D in high density (due to probably suboptimal auxin signaling) had provoked an inadequate immune system response, resulting in the slowdown of growth of bean plants in comparison with inoculation with another dose–strain combination. Although all growth indicators of the 7-day-old plants, inoculated with 26D strain (dose 10^8^ cells mL^−1^), were at the control level (Figure 1), the MDA content in the roots was significantly higher than the control (Figure 2), which indicated an accumulation of lipid peroxidation, caused, possibly, by a high pool of ROS. At the same time, the accumulation of H_2_O_2_ in plant tissues (especially in shoots) of ineffective variant of inoculation (26D strain, dose 10^8^ cells mL^−1^) was very low (Figure 3a). This could be explained either by the low generation of hydrogen peroxide or its effective utilization. The absolute values of oxidoreductases activity in plants did not allow us to see the specific features of the ineffective inoculation variant. Only the recalculation of enzyme activity per unit of H_2_O_2_ contained in tissues appeared to have a low content of H_2_O_2_ in the ineffective variant of inoculation (26D strain, dose 10^8^ cells mL^−1^), which was the result of the intense work of oxidoreductases (Table 6).

It must be admitted that the role of the plant immune system in detecting and controlling pathogenic microorganisms has been well described, but much less is known about the immune response towards the wealth of commensals that inhabit plants [54,55]. Upon recognition of microbial molecules, the first layer of immunity, known as pattern-triggered immunity (PTI), plasma membrane-localized pattern recognition receptors (PRRs) recognize microbe-associated molecular patterns (MAMPs) and trigger various immune responses, such as the production of ROS, calcium influx, MAP kinase activation, transcriptional reprogramming, and production of defense phytohormones and specialized metabolites [56]. Usually, non-pathogenic microbes are thought to trigger only a weak immune response [57]. In mutualistic interactions, most regulatory pathways are associated with disabling protective pathways that would otherwise prevent fungal proliferation or bacteria in plant tissue [58]. Obviously non-pathogenic members of the root microbiome actively interfere with plant immune signaling either evade PRR-mediated immune recognition or interfere with the subsequent immune signaling process, also through the delivery of immune-suppressive effector molecules, which can efficiently perturb plant immunity by eliminating the transient ROS burst or by means of modulating the effect on hormonal signaling [59].

One prominent PTI output involves activation of the plasma membrane-localized NADPH oxidase respiratory burst oxidase homolog D (RBOHD), producing the ROS in the extracellular space, which can then be readily converted to H_2_O_2_ via SOD in the apoplast [56]. Being regulated by several different factors, NADPH oxidase was shown to play a significant role in mutualistic and symbiotic processes between plant and microorganism [60,61]. In order to successfully colonize rice roots, the PGPB Gluconacetobacter diazotrophicus required the up-regulating of the transcript levels of ROS-detoxifing genes, such as SOD and glutathione reductase (GR) [62]. Apparently, the mutualistic nature of the relationship between endophytic bacteria and the plant should be characterized by a balance of generation and detoxification of ROS.

One of the products of ROS neutralization is H_2_O_2_, it is the most stable form of activated oxygen, due to its low reactivity and lack of charge; it capable of spreading over significant distances and is considered as an intra-cellular messenger [63,64]. In experiments on the inoculation of beans with growth-stimulating EB under normal conditions and under salinity, a large variability in the H_2_O_2_ content in roots and shoots, as well as the activity of antioxidant enzymes, was noted depending on the strain; in particular, in the tissues of the roots and shoots of plants inoculated with the strain HR26, which resulted in the greatest dry weight of roots and shoots, the increased level of accumulation of H_2_O_2_ was noted [65]. This is consistent with the high levels of H_2_O_2_ in plant tissues of 7-day plants with the best growth traits (Figure 3a).

To predict the effectiveness of symbiotic relationships, it is useful to have indicators of the physiological and biochemical state of plants, reflecting the formation of mutualistic relationships between the genotype of a variety and strain, in order to use the identified markers for the selection of complementary strain–dose combinations to plant genotype. In the dry years of 2018 and 2023, when there was a positive effect of inoculation, the content of H_2_O_2_ in roots of the inoculated 7-day plants positively correlated with the final seed productivities (0.89 and 0.77). But the coefficients were zero and negative (−0.04 and −0.52) in less stressful conditions (2020 and 2021), when inoculation didn’t result in the increase of seed productivity. Therefore, it can be assumed that in milder agroclimatic conditions, plants rely more on their own resources without entering into mutualistic relationships with endophytic bacteria. At the same time CAT activity and MDA content in the roots of week-old plants negatively closely (−0.99 and −0.98) correlate with the final seed productivity of inoculated plants over all experiments in the four-year period. The totality of this data may mean that the effective inoculation was accompanied by a weakening of catalase activity in the roots of the 7-day plants, leading to the accumulation of H_2_O_2_ and the minimization of lipid peroxidation in the roots. These processes could be considered to be a consequence of the participation of bacteria in the rhizophagy cycle, described above. And the effects observed in the variant of inoculation with strain 26D at a dose of 10^8^ cells mL^−1^, namely a reduced level of H_2_O_2_ and the accumulation of MDA, indicate that the processes occurring in plants could correspond to the scenario of suppression of the rhizophagy cycle. It is important to note that only a high dose of this strain caused this effect, therefore, the factor that caused it can be corrected by adding a lower dose.

Previously, it was proposed to use a decrease in the level of MDA in inoculated 7-d-old seedlings compared to the control as a biochemical marker of effective variety–strain combinations in model experiments under controlled conditions [66]. In this study, the relationship between the decrease in MDA content in the roots of 7-day-old inoculated plants in vitro and their ability to increase the seed yield in a field experiment was confirmed. In addition, in this study, specific values of enzyme activity per unit of H_2_O_2_ content were used to analyze correlations. The correlation relationships between recalculated values of each oxidoreductase activity per unite H_2_O_2_ content were closer than the absolute values of the activity of each enzyme. Further studies are expected to shed light on the mechanisms behind these facts.

## 4. Materials and Methods

### 4.1. Plant Material and Bacterial Strains

The studies were carried out on common bean plants (*Phaseolus vulgaris* L., Ufimskaya cultivar). The seeds were initially purchased from the originator of the cultivar, prof. S. Samigullin, and then were reproduced by prof. S. Garipova in a multi-year reproduction of scientific studies [33,35,45].

EB for inoculation were obtained from the collection of the Bashkir Research Institute of Agriculture, UFSC RAS: strain *B. subtilis* 26D (VKPM No. 016-02-2491-1) and strain *B. subtilis* 10–4 (VKPM V-12988).

### 4.2. Bacterial Inoculants Preparation

In the collection, strains were stored on solid potato–glucose agar on slanted surfaces in test tubes in a refrigerator at 4 °C. Bacterial biomass from a museum tube was washed and homogenizing with sterile water using Vortex and 1 mL of a suspension of a spore culture of bacteria was inoculated to the liquid potato–glucose medium. A total of 200 g of peeled, finely chopped potatoes were poured into 1 L of tap water and boiled for 30 min. The filtered broth was added with water to a liter, then added glucose 10 g L^−1^ and then sterilized and left for 30 min at 1.5 atm. The cultivation of the liquid culture of bacteria was carried out in Erlenmeyer flasks in a thermostated shaker (Water bath shaker clpnn type 357, Lubawa, Poland) at a temperature of 30 °C for 72 h at 150 RPM. The titer of the resulting drug was 10^10^ cells mL^−1^. The model concentration of bacteria (10^9^ cells/mL) was created according to the Tarasevich turbidity standard (Scientific Center for Expertise and Medical Applications, Moscow, Russia). The specified density of bacterial cells (10^5^ and 10^8^ cells/mL) was obtained by the method of 10-fold dilutions. The homogeneity of the intermediate dilutions suspension was generated using the Multi-Vortex V-32 device (BioSan, Rīga, Latvia). The density of bacterial cells in the suspension was additionally verified, counting the number of cells in a Goryaev chamber (MiniMed, Bryansk, Russia).

### 4.3. Seed Inoculation

For the small-scale field experiments in 2018, 2020, and 2021, wanting to bring the inoculation method closer to production conditions, the seeds were inoculated using a semi-dry method by shaking them in bacterial suspensions (10 µL g^−1^ of seeds) for 4 min. In 2023, in order to ensure reliable colonization of seeds with bacteria, the seeds were inoculated by soaking the seeds in a suspension of bacterial cells for 3 h (1 mL g^−1^ of seeds). Previously, the ability of bacteria to colonize bean seedlings was tested after 1 h of soaking [40]. The seeds similarly treated with sterile water served as a control. The seeds were sown within 18 h after inoculation.

*For laboratory experiments*, the seeds were sterilized with a Brilliant disinfectant (alkyldime-thylbenzylammonium chloride 0.9%, glutaraldehyde 0.8%) for 10 min and washed repeatedly with distilled water. A total of 20 bean seeds of each experimental variant were soaked in 4 mL of the bacterial suspensions in Petri dishes for 3 h, and then placed on moistened filters in plastic containers (size 15 × 20 × 15 cm) with lids. In the control, the seeds were soaked in sterile water. The seeds were germinated in the dark at a temperature of 22–24 °C.

### 4.4. Small-Scale Field Experiments

The field experiments were carried out in 2018, 2020, 2021, and 2023 in four regions of the Southern Ural (Republic of Bashkortostan, Russia): the Salavatsky region in 2018; the Ilishevsky region in 2020; the Arkhangelsky region in 2021; and in the Iglinsky region in 2023. The agroclimatic and soil conditions of the experimental plots are given in Table 8. The growing season of 2018 (HTC 0.63), against the background of the predominance of hot and dry weather, was characterized by frosts, snowfall in mid-June, and cold temperatures in September, which shortened the duration of the bean ripening period by two to three weeks. In 2020 (HTC 1.14), the growing season was characterized as relatively favorable and in the most vulnerable phases of plant development from germination to flowering, a fairly rapid accumulation of effective temperatures was recorded with an optimal precipitation ratio. Under the conditions of 2021 (HTC 0.95), soil drying was noted in the spring and a significant increase in temperature indicators during the daytime hours during the growing season (May–August). In 2023 (HTC 0.47), during the spring–summer period, increased average daily temperatures were observed with insufficient precipitation. Sowing of beans was carried out manually using a wide-row method with a row spacing of 45 cm and a seeding rate of 15 viable seeds per 1 running meter. (333 thousand units ha^−1^), and in 2023, the row spacing was 30 cm. Sowing time was the third ten days of May. Cleaning dates: the first ten days of September. 

#### 4.4.1. Determination of Plant Growth and Productivity Parameters

In 2023 the germination was determined at the 9th (seedling stage) and 38th day (budding—beginning of flowering) after sowing, survival—at the 120th day, at harvesting. The percentage of sprouted or surviving plants of number of sown seeds (*n* = 60) was taken into account. Plant height was estimated from the soil surface to the petiole of the topmost of the leaf; 60 plants were measured. To analyze the rate of development, each of the 60 plants was assessed and the number of plants that passed into the next phase of development was noted. Each plant was counted once and indicated by the presence of the corresponding signs: the appearance of buds (budding stage), flowers (flowering stage), fruits (pods appearance), thickening of pods (beginning of maturation), and full ripening (drying of fruits and hardening of seeds). The seed productivity and the structure of the crop was analyzed based on the biometric indicators of 15 individual plants: number of pods, number of seeds, and mass of seeds per plant. The mass of 1000 seeds was calculated by means of weighing three replicates of 100 seeds from each variant, taking the arithmetic mean and multiplying by 10.

#### 4.4.2. Determination of Water Content in Plant Leaves

Bean leaf samples were taken at the beginning of ripening, dried to a constant weight with periodic weighing after 6, 24, and 48 h [67,68]. Fresh weight was determined immediately after the leaves were separated from the plant. To determine the turgor mass, the leaves were placed in containers with water for 6 h. Total water content (W), water holding capacity (R), and relative water content (RWC) in samples were calculated using the following formulas:

W = 100 · (M − M2)/M,

R = 100 · [(M − M2) − (M − M1)]/M = 100·(M1 − M2)/M,

RWC = [(M − M2)/(TM − M2)] · 100

where M is the mass of the fresh sample; M1—sample weight after 24 h of drying; M2—sample weight after 48 h of drying; and TM—mass at full turgor.

### 4.5. Laboratory Experiments

#### 4.5.1. Determination of Seedlings Growth

Growth parameters were assessed on 20 plants with a 7-day-old age. The growth parameters included the length of primary root and shoot, number and length of adventitious roots, and root and shoot fresh biomass.

#### 4.5.2. Determination of Catalase (CAT) Activity

Catalase (CAT, EC 1.11.1.6) activity was determined by measuring the decomposition of H_2_O_2_, as described [69]. After homogenizing the plant tissues with 0.05 M potassium phosphate buffer at pH 7.8, based on the ratio 1:5 (m:v), the mixture was centrifuged at 13,000× *g* for 10 min at 4 °C. The reaction was started by adding 0.15 mL of 0.03% H_2_O_2_ to the 0.02 mL enzyme extract in the wells of a flat bottom plate for immunoassay (Costar, Washington, DC, USA). After 1 min, the reaction was terminated by adding 0.075 µL 4% (NH_4_)6Mo_7_O_24_ × 4H_2_O. CAT activity was measured by a Benchmark microplate reader (Bio-Rad, Hercules, CA, USA) as changing the absorbance at 405 nm due to the extinction of complexes of H_2_O_2_ with molybdenum ions.

#### 4.5.3. Determination of Peroxidase (POD) Activity

Peroxidase (POD, EC: 1.11.1.7) activity was assayed as described by [70], scanning the ability of enzyme extract’s to prevent o-phenylenediamine (OPD) oxidation. The plant tissue was homogenized in a 0.01 M potassium phosphate buffer at pH 7.8, based on the ratio 1:5 (m:v). The extracts were centrifuged at 13,000× *g* for 10 min at 4 °C. The POD activity was assayed by a micromethod in wells of a flat bottom plate for immunoassay (Costar, Washington, DC, USA). The reaction was initiated by the addition of the plant extract for 1 min, followed by a reaction termination with the addition of 4N H_2_SO_4_. Samples were measured by a Benchmark microplate reader (Bio-Rad, Hercules, CA, USA) at 492 nm.

#### 4.5.4. Determination of Superoxide Dismutase (SOD) Activity

Superoxide dismutase (SOD, EC 1.15.1.1) activity was measured in wells of a flat bottom plate for immunoassay (Costar, Washington, DC, USA) by spectrophotometer EnSpire 2300 (Perkin Elmer, Waltham, MA, USA) at 540 nm according to Beyer and Fridovich [71]. The plant tissues were homogenized in a 0.01 M potassium phosphate buffer at pH 7.8, based on the ratio 1:10 (m:v). The mixture was centrifuged at 13,000× *g* for 10 min at 4 °C. The SOD activity was assayed by measuring its ability to inhibit the photochemical reduction of nitro blue tetrazolium (NBT, Merk, Germany). For the analysis, a 10 µL enzyme extract was mixed with a 200 µL reagent I (0.15 M potassium phosphate buffer at pH 7.8, containing 0.1 mM of EGTA, 0.6 mM of NBT, and 0.5 mM of phenazine methasulfate) and 10 µL reagent II (150 mM Tris-EDTA buffer at pH 8.0, containing 0.1 mM of NADH). Absorbances of the control (10 µL extraction buffer mixed with 200 µL reagent I and 10 µL reagent II) were considered in the assay. One unit of the SOD activity (U) was defined as the amount of enzyme required to result in a 50% inhibition of the rate of nitro blue tetrazolium reduction at 540 nm. Results were given as a specific enzyme activity unit (mg protein × min)^−1^. Total protein content was quantified according to the Bradford method [72], in wells of a flat bottom plate for immunoassay (Costar, Washington, USA), with chymotrypsin as the standard by spectrophotometer EnSpire 2300 (Perkin Elmer, Waltham, MA, USA) at 595 nm.

#### 4.5.5. Determination of Hydrogen Peroxide (H_2_O_2_) Content

Hydrogen peroxide (H_2_O_2_) was determined in plant extracts according to [73]. The plant tissues were homogenized in a 0.01 M potassium phosphate buffer at pH 6.2, based on the ratio 1:5 (m:v), extracted for 10–15 min at 4 °C. The extracts were centrifuged at 10,000× *g* for 10 min at 4 °C and 50 μL of the supernatant was used to initiate the reaction in a mixture (total volume of 150 μL) containing 25 mM of FeSO_4_ and 25 mM of (NH_4_)2SO_4_ that were dissolved in 2.5 M of H_2_SO_4_. One mL of this solution was added to 100 mL of 125 μM xylenol orange (Merk, Darmstadt, Germany) and 100 mM sorbitol. The H_2_O_2_ content was monitored with xylenol orange dye; when hydroperoxides are reduced by ferrous ions in an acidic solution, they form a ferric product–xylenol orange complex. The reaction was incubated in darkness at room temperature for 1 h and absorbance was recorded by spectrophotometer EnSpire 2300 (Perkin Elmer, Waltham, MA, USA) at 570 nm. The H_2_O_2_ content was calculated using a standard curve based on the absorbance of H_2_O_2_ standards.

#### 4.5.6. Determination of Malondialdehyde (MDA) Content

Malondialdehyde (MDA) content was determined by measuring its reaction with the thiobarbituric acid [74]. The frozen 0.25 g of plant tissues were homogenized with 3 v of ice cold 10% trichloroacetic acid (TCA), and centrifuged at 10,000× *g* for 15 min. An assay mixture containing 1 mL aliquot of the supernatant and 1 mL of 0.5% (*m*/*v*) 2-thiobarbituric acid in 20% (m:v) TCA was heated to 95 °C for 1 h and then rapidly cooled in an ice-bath. The absorbance of supernatant was monitored at 532 nm and 600 nm using a spectrophometer Smart Spec Plus (Bio-Rad, Hercules, CA, USA). MDA content was calculated using 155 mM^−1^ cm^−1^ as a coefficient of absorbance.

### 4.6. Statistical Analysis

The experiments were carried out in three biological and three analytical replicates. A statistical analysis package in Microsoft Office Excel 2010 was used for the analysis of variance (ANOVA) between the treatment groups. Data presented were mean values with standard errors (±SE).

## 5. Conclusions

The finding based on the average values of seed productivity (SP) in the four-year field experiments demonstrated that MDA content, as well as CAT activity in the roots of week-old plants in vitro, were negatively closely correlated with the final SP of the inoculated plants. The weakening of CAT activity naturally led to the accumulation of H_2_O_2_ in the roots. But high positive correlation coefficients between the content of H_2_O_2_ in the roots, as well as the length of root in the initial stages of symbiosis formation in vitro, and the final field SP were traced only in arid hydrothermal conditions, when inoculation with strain 10–4 led to a significant effect on yield. The SP of inoculated plants was positively correlated with root length and negatively correlated with MDA content and catalase (CAT) activity in roots; at the same time, SP positively correlated with H_2_O_2_ content and CAT activity in shoots. The physiological indicators, such as the increase in root length and decrease in MDA content and CAT activity in roots, as well as the increase of H_2_O_2_ content and CAT activity in shoots, are considered as potential markers for predicting the effectiveness of the endophytic symbiosis between bean plants and *B. subtilis* (strains 10–4 and 26D). The obtained results will be useful in developing future eco-friendly technologies to improve bean cultivation.

## Figures and Tables

**Table 1:** Seed productivity of bean plants when treated with *B. subtilis* strains 26D and 10–4 in small-scale field studies in 2018–2023 upon different hydrothermal coefficient (HTC).

Strain	Dose (Cells mL^−1^)	2018 (HTC 0.63)		2020 (HTC 1.14)		2021 (HTC 0.95)		2023 (HTC 0.47)		Average (% to Control)	
		1 *	2 **	1 *	2 **	1 *	2 **	1 *	2 **	1 *	2 **
Control	-	103 ± 1.1 b	4.0 ± 0.5 b	566 ± 4.4 b	12.3 ± 0.8 b	187 ± 3.4 b	8.4 ± 0.8 a	200 ± 2.3 b	3.6 ± 0.5 a	100 ± 2.8 b	100 ± 0.6 b
26D	10^5^	-	-	450 ± 4.7 a	9.0 ± 0.5 a	-	-	155 ± 2.6 a	3.6 ± 0.7 a	79 ± 3.6 a	87 ± 0.6 a
26D	10^8^	83 ± 2.7 a	3.4 ± 0.5 a	-	-	164 ± 2.6 a	7.7 ± 0.9 a	143 ± 2.5 a	3.1 ± 0.6 a	80 ± 2.6 a	88± 0.7 a
10–4	10^5^	123 ± 2.3 c	5.2 ± 0.7 c	516 ± 5.8 a	11.0 ± 0.6 b	181 ± 3.1 b	7.8 ± 0.8 a	240 ± 4.3 c	5.1 ± 1.3 b	107 ± 3.8 b	114 ± 0.9 c
10–4	10^8^	-	-	-	-	-	-	204 ± 3.9 b	5.7 ± 1.0 b	102 ± 3.9 b	158 ± 1.0 d

* Seed productivity per square meter (g), values are the mean of 3 replicates; ** seed productivity per plant (g), values are the mean of 15 plants. Different letters show significant difference at *p* ≤ 0.05.

**Table 2:** Morphometric parameters of beans when treated with *B. subtilis* strains 26D and 10–4 in small-scale field studies in 2023 (HTC 0.5).

Strain	Dose (Cells mL^−1^)	Germination (%)		Survival (%)	Height of Shoot (cm)	
		9th Day	38th Day	120th Day	38th Day	120th Day
Control	-	47 a	67 a	70 a	14.0 a	39.8 a
26D	10^5^	58 ab	80 b	80 ab	14.4 a	38.8 a
26D	10^8^	57 ab	83 b	75 a	15.3 ab	41.9 ab
10–4	10^5^	73 b	90 c	87 b	16.4 b	42.7 b
10–4	10^8^	68 b	77 b	80 ab	14.6 a	42.7 b

Values are the mean of 60 plants ± SE. Different letters show significant difference at *p* ≤ 0.05.

**Table 3:** Proportion (%) of bean plants that have entered a certain phase of the growing season, when treated with *B. subtilis* strains 26D and 10–4 in small-scale field studies in 2023.

Strain	Dose (Cells mL^−1^)	38th Day of Growing Season		120th Day of Growing Season		
		Budding	Flowering	Pods Appearance	Beginning of Maturation	Full Ripeness
Control	-	60	40	33	40	27
26D	10^5^	39	58	27	27	46
26D	10^8^	33	67	7	13	80
10–4	10^5^	23	76	13	7	80
10–4	10^8^	17	83	7	20	73

**Table 4:** Water content in leaves of bean plants when treated with *B. subtilis* strains 26D and 10–4 in small-scale field studies in 2023.

Strain	Dose (Cells mL^−1^)	W (%)	R (%)	RWC (%)
Control	-	83.3 ± 1.2 a	23.1 ± 1.1 a	37.7 ± 3.2 b
26D	10^5^	82.9 ± 0.7 a	43.8 ± 2.0 d	31.3 ± 2.8 a
26D	10^8^	84.0 ± 0.4 a	31.7 ± 2.5 b	44.3 ± 0.9 c
10–4	10^5^	81.1 ± 0.7 a	34.0 ± 1.4 bc	41.2 ± 1.1 bc
10–4	10^8^	81.6 ± 0.8 a	36.6 ± 2.2 c	42.8 ± 1.4 c

W—total water content, R—water holding capacity, RWC—relative water content. Values are the mean of 3 leaves in 5 replicates. Different letters show significant difference at *p* ≤ 0.05.

**Table 5:** Yield structure of bean plants when treated with *B. subtilis* strains 26D and 10–4 in field studies in 2023.

Strain	Dose (Cells mL^−1^)	Number of Pods per Plant (pcs)	Number of Seeds per Plant (pcs)	Seed Weight per Plant (g)	Weight of 1000 Seeds (g)
Control	-	6.1 ± 0.7 b	13.6 ± 2.0 a	3.6 ± 0.5 a	265 ± 0.7 b
26D	10^5^	5.3 ± 0.7 a	12.1 ± 2.2 a	3.6 ± 0.7 a	298 ± 1.1 c
26D	10^8^	5.4 ± 0.9 a	14.2 ±2.3 a	3.1 ± 0.6 a	218 ± 1.3 a
10–4	10^5^	8.1 ± 1.3 c	18.3 ± 4.0 ab	5.1 ± 1.3 b	279 ± 0.9 bc
10–4	10^8^	8.5 ± 1.3 c	22.5 ± 3.6 b	5.7 ± 1.0 b	253 ± 1.0 b

Values of yield structure are the mean of 15 plants. Different letters show significant difference at *p* ≤ 0.05.

**Table 6:** Coefficients of oxidoreductase activity in terms of the level of hydrogen peroxide (H_2_O_2_) content in the tissues of inoculated plants.

Strain	Dose (Cells mL^−1^)	SOD/H_2_O_2_		CAT/H_2_O_2_		POD/H_2_O_2_	
		Roots	Shoots	Roots	Shoots	Roots	Shoots
Control	-	2.76 b	2.21 b	0.15 b	0.12 b	0.29 b	0.12 a
26D	10^5^	1.42 a	1.04 a	0.15 b	0.16 c	0.25 a	0.12 a
26D	10^8^	3.86 c	2.82 c	0.23 b	0.23 d	0.34 c	0.23 b
10–4	10^5^	1.78 a	0.83 a	0.10 a	0.09 a	0.24 a	0.08 a
10–4	10^8^	3.12 b	1.12 a	0.11 a	0.17 c	0.23 a	0.27 b
LSD_05_		0.44	0.37	0.02	0.02	0.02	0.04

Different letters show significant difference at *p* ≤ 0.05.

**Table 7:** Correlation coefficients of bean productivity per plant in various agroecological conditions with morphometric and physiological-biochemical parameters of plants in model conditions.

Plant Trait	2018	2023	2020	2021	Average in 4 Years
	HTC 0.6–0.5		HTC 1.1–0.9		
Shoot height	0.98	0.62	0.55	0.07	0.90
Root length	0.99	0.94	0.43	−0.06	0.83
Number of adventitious roots	0.99	0.30	0.37	−0.14	0.79
Length of adventitious roots	−0.69	0.52	−0.95	−0.70	−0.98
Plant weight	0.99	0.49	0.33	−0.17	0.76
Shoot mass (fresh weight)	0.74	0.39	−0.30	−0.73	0.22
Root mass (fresh weight)	0.88	0.56	−0.07	−0.55	0.44
POD activity in the roots	0.03	0.32	−0.89	−0.99	−0.55
POD activity in the shoots	0.82	0.94	−0.18	−0.64	0.34
CAT activity in the root	−0.74	−0.50	−0.92	−0.62	−0.99
CAT activity in shoots	0.86	0.60	−0.10	−0.58	0.41
H_2_O_2_ content in the roots	0.89	0.77	−0.04	−0.52	0.47
H_2_O_2_ content in the shoots	0.98	0.56	0.29	−0.22	0.73
SOD activity in the root	−0.78	0.44	−0.90	−0.57	−0.99
SOD activity in the shoot	0.91	0.10	0.75	0.33	0.98
MDA content at the root	−0.71	−0.84	−0.94	−0.65	−0.98
MDA content at the shoot	−0.98	−0.27	−0.23	0.28	−0.69
SOD activity/H_2_O_2_ in the root	−0.97	−0.16	−0.63	−0.16	−0.93
SOD activity/H_2_O_2_ in the shoot	−0.99	−0.71	−0.41	0.09	−0.82
CAT activity/H_2_O_2_ in the root	−0.94	−0.84	−0.70	−0.26	−0.96
CAT activity/H_2_O_2_ in the shoot	−0.86	−0.42	−0.82	−0.43	−0.99
POD activity/H_2_O_2_ in the root	−0.98	−0.81	−0.60	−0.13	−0.92
POD activity/H_2_O_2_ in the shoot	−0.89	0.17	−0.79	−0.38	−0.99

**Table 8:** Soil and climatic conditions of small-plot field experiments.

Indicators	2018	2020	2021	2023
Region of Bashkortostan Rupublic	Salavatsky	Ilishevsky	Arkhangelsky	Iglinsky
HTC_(VI–IX)_	0.63	1.14	0.95	0.47
Σ T_effect>15_ (°C)	414.3	351.5	491.4	371.9
R (ᴍᴍ)	34.5	68	45	25.5
Soil type	Gray forest	Leached chernozem	Gray forest	Gray forest
Humus (%)	3.1	7.6	4.1	4.4
Alkaline hydrolyzable nitrogen (mg kg^−1^)	93	156	105	122
Mobile phosphorus (mg kg^−1^)	28	109	45	51
pH H_2_O	5.8	6.8	5.2	5.9

## Data Availability

The data presented in this study are available on request from the corresponding author.
